# An insight into immunogenic salivary proteins of *Anopheles gambiae *in African children

**DOI:** 10.1186/1475-2875-6-75

**Published:** 2007-06-05

**Authors:** Sylvie Cornelie, Franck Remoue, Souleymane Doucoure, Tofene NDiaye, Francois-Xavier Sauvage, Denis Boulanger, Francois Simondon

**Affiliations:** 1 Unité de Recherche Epidemiologie et Prevention (UR024), Centre IRD de Montpellier, BP 64501,911 avenue Agropolis, 34394 Montpellier cedex 5, France; 2 Unité de Recherche Epidemiologie et Prevention (UR024), Campus IRD de Hann, BP1386, Route des Pères Maristes, CP 18524, Dakar, Sénégal; 3 UMR Sciences pour l'Œnologie, INRA Centre de Montpellier, 2 place Viala 34060 Montpellier cedex 1, France

## Abstract

**Background:**

During blood feeding, the mosquito injects saliva into the vertebrate host. This saliva contains bioactive components which may play a role in pathogen transmission and in host-vector relationships by inducing an immune response in the vertebrate host. The evaluation of human immune responses to arthropod bites might also represent a research direction for assessing individual exposure to the bite of a malaria vector.

**Methods:**

The present study examined the antibody (Ab) IgG response during the season of exposure to *Anopheles gambiae *bites in young children living in a malaria endemic area. Immunoblots were performed with *An. gambiae *saliva to detect anti-saliva Ab bands and the evolution of immunogenic bands at the peak of, and following, the transmission period.

**Results:**

The results showed that anti-*Anopheles *Ab was directed against a limited number of salivary proteins (175, 115, 72 and 30 kDa bands). Specific IgG responses to mosquito salivary proteins were variable among exposed individuals; nevertheless, two major bands (175 and 72 kDa) were observed in all immune-responder children. Analysis of the intensity of immunogenic bands revealed that IgG levels against the 175 kDa band were significantly higher during the peak period compared to the end period malaria transmission.

**Conclusion:**

This preliminary work supports the potential of using anti-saliva immune responses as a measure of exposure to *Anopheles *bites. The use of immunoblots coupled with evaluation of band intensity could be an adequate tool for distinguishing immunogenic salivary proteins as candidate markers of bite exposure. Furthermore, this study may open the way to design new epidemiological tools for evaluating the risk of malaria exposure.

## Background

Morbidity and mortality of malaria are closely linked to exposure of the human host to the *Anopheles *vector. Among the 60 *Anopheles *species which transmit malaria worldwide, *Anopheles gambiae *is the most competent and the primary vector of *Plasmodium falciparum *in sub-Saharan Africa [1]. Human infection by *P. falciparum *can be measured by several diagnostic tests, such as thick smears or rapid diagnostic tests (RDT, Dipstick) [2]. The evaluation of exposure to risk of malaria is currently based on entomological methods (traps, household/indoor spraying, etc.), but such methods are mainly applicable to the population level and do not enable evaluation of the heterogeneity of individual exposure. Trapping methods using adults volunteers can estimate individual exposure, but may be limited due to ethical constraints and limitations for extrapolation to the incidence in children [3].

During the bite, the female mosquito injects saliva containing bioactive molecules such as vasodilatators, anti-clotting and anti-hemostatic proteins, which permit a successful blood meal [4]. During the last decade, research on mosquito salivary extracts has benefited from advances in genomic and proteomic tools generating salivary transcriptomes for several mosquito species, including *An. gambiae *[5-8]. In particular, interactions between salivary proteins and the host immune system have aroused great interest [9,10]. Indeed, some of these salivary components are recognized by the human immune system and induce specific responses [11]. Interestingly, a connection between the level of specific saliva antibodies (Ab) and exposure to vector borne-diseases has been reported for Lyme disease, Chagas disease, leishmaniasis [12-14], and recently, human African trypanosomiasis [15]. Surprisingly, few data have described an immunomodulatory role for salivary proteins from *Anopheles *species and little is known about the human host response to *Anopheles *saliva. The Ab response to *Anopheles stephensi *saliva has been detected in allergic individuals [16]. Owashi and colleagues identified an inflammatory protein from *An. stephensi *saliva, which is recognized by sera from individuals exposed to malaria [17]. A recent report indicated that the IgG response to whole saliva from *An. gambiae *was found in children living in a malaria endemic area and was correlated with the intensity of exposure to *An. gambiae *bites [18]. That study led to the hypothesis that this specific Ab response could represent a marker of exposure to *Anopheles *bite. Thus, the objective of the present work was to describe immunogenic proteins of *An. gambiae *saliva so as to elucidate whether detection of immunogenic components depends upon the malaria transmission season.

## Methods

### Studied population

The present study was carried out in Niakhar, a rural area of Senegal situated 115 km east of Dakar. This site is a dry savannah with a rainy season from July to October. The region is typical of the Sahel and sub-Sahel regions of Africa, where the occurrence of malaria is unstable, with a season of *P. falciparum *transmission from September to November [19]. Sera were available from a large clinical trial on intermittent preventive treatment against malaria performed in 1,200 children in 2002 [20]. Sera from a subsample of these children aged from 5 weeks to 35 months (n = 30) were available at the peak (September) and at the end (December) of the malaria season. The present study adhered to the ethical principles of the Edinburgh revision of the Helsinki Declaration, and was approved by the ethical committees of Senegal and the IRD (August 2002, January 2004, respectively). Informed consent was obtained from the parents of included children. Control sera were obtained from healthy adults living in a non-malaria region in Montpellier, France [15].

### Reagents

All water used was of 18 MΩ quality and was produced using an EASYpure^®^II apparatus (Barnstead, Dubuque, Iowa, USA).

### Mosquito saliva

Adult female *An. gambiae *s.s. were bred in an insectarium in Dakar (IRD-UR77). Saliva from 10- to 20-day-old uninfected *An. gambiae *females was collected as previously described [18,21]. Saliva in buffer (Hepes/NaCl/EDTA, pH7.2) was stored at -80°C before use. A large pool of saliva (n = 384 mosquitoes) was constituted for all experiments. Protein concentrations were measured by the bicinchoninic acid method (BCA, Pierce, Rockford, IL, USA).

### Electrophoresis and staining

One-dimensional electrophoresis (1-DE) was carried out using Criterion XT- Bis-Tris 4 to 12% acrylamide gels (Biorad, Marnes-la -Coquette, France). Saliva from 48 mosquitoes was loaded on each gel and run with XT-MES buffer according to the manufacturer's instructions. Gels were silver-stained as described by Poinsignon et al. [15].

### Immunoblot

Gels were transferred onto a PVDF membrane (polyvinyldifluoride, Biorad) for 2 h, 400 mA. Free binding sites on the membrane were blocked by incubation in 5% dry milk dissolved in TBS for 45 min at room temperature. After washing three times with TBS Tween 0.1% (TBST), membranes were incubated overnight at 4°C with serum samples diluted 1/100 in blocking buffer. The membranes were then incubated with alkaline-phosphatase mouse monoclonal anti-human IgG antibody at a dilution of 1/5000 (Sigma, St Louis, MO) for 2 h at room temperature after three washes in TBST. The membranes were revealed with chemoluminescent substrate (Lumi-Phos WB chemoluminescent substrate, Pierce) and exposed to CL-Xposure film (Pierce) for 5 s. Prestained standards (Fermentas, Burlingtion, Ontario, Canada) were used to determine the relative molecular weights of the electrophoresed components. The peak and post-season serum samples of each subject were consistently examined in the same immunoblot experiment, and a positive control serum (pool of responder children) was used for each immunoblot. The films were scanned by an "Image scanner II" (GE healthcare, Uppsala, Sweden) and the intensities of immunogenic bands were analysed by the Scion Image Program (Scion Corporation). A numeric value was calculated for each band by analysing the mean intensity of staining. Results represented a ratio sample band/standard band and were expressed as integrated OD (iOD).

### Statistical analysis

Data were analysed with a Graph Pad Prism^® ^(Graphpad Software Inc). After verifying that values in each group did not assume a Gaussian distribution, the non-parametric Wilcoxon matched pair was used to compare the intensity of immunogenic bands between September and December. Differences were considered significant at P < 0.05.

## Results

### Salivary proteins of the *Anopheles gambiae *mosquito

**Figure 1:**
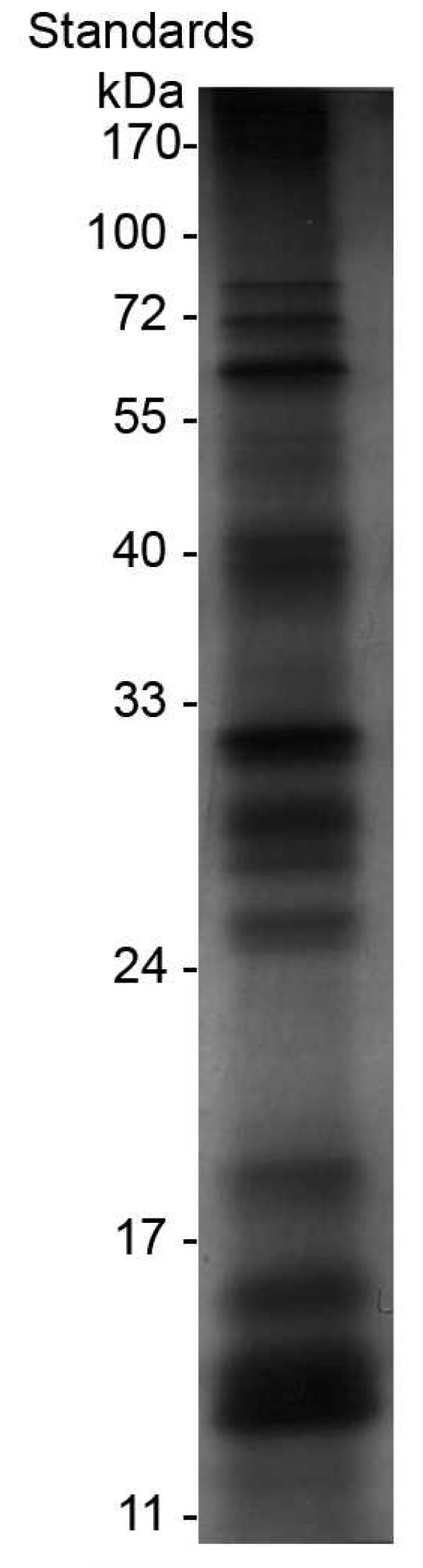
**Protein content of *An. gambiae *saliva**. Saliva from 4 mosquitoes was run on a large 12% acrylamide gel under non-denaturing conditions (right lane) and stained with silver nitrate. Molecular weight markers are shown in the left lane.

Electrophoresis was carried out to profile the protein content of saliva from *An. gambiae*, which showed numerous bands ranking from 10 kDa to > 100 kDa (Figure 1). About twenty polypeptides with molecular masses of 103, 94, 74, 62, 54, 49, 41, 39, 31, 30, 28, 26, 24, 16, 14, 13.5 and 12 kDa were clearly detected in silver-stained gel and a few others were lightly stained. Several batches of saliva were tested with similar results.

### Identification of immunogenic proteins in sera of young children

**Figure 2:**
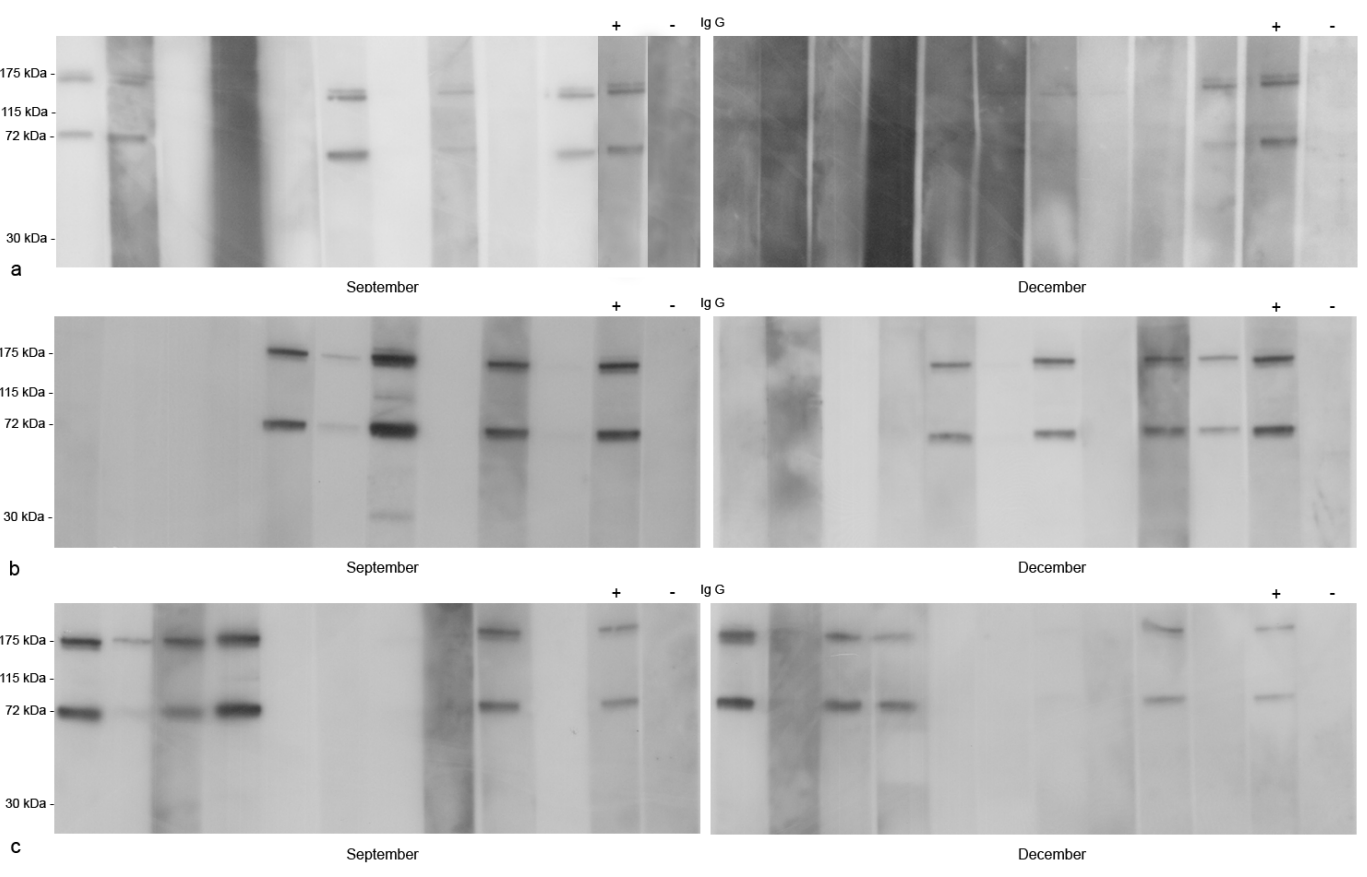
**IgG Immunoblots of anti-saliva antibodies to *An. gambiae *in peak and postseason sera of African children**. 30 sera from children aged to 5 weeks to 35 months were tested from IgG anti-saliva antibodies during the peak and post- malaria transmission seasons. 3 series of two gels representing 10 individuals each were performed (a, b, c). The peak season blots show intense 175- and 72- kDa antibody bands. Lane +: positive control, pool of sera showing a positive response in ELISA [18]. Lane -: negative control from people living in non-malaria endemic area.

IgG immunoblots were performed with 30 sera from the same young children in September (peak) and December (the end of the malaria transmission season). Immunoblots revealed substantial individual variations in intensity and number of detected immunogenic proteins. The IgG Abs reacted with a few salivary proteins with molecular masses of 175, 115, 72 and 30 kDa (Figure 2). Some of these were the minor components of saliva which were not clearly visible in silver-stained gels. 53% of the children were positive in September and 43% in December; 40% of the children show immunogenic bands either in September and in December. In the positive children, two major immunogenic bands (175 and 72 kDa) were consistently observed. Sera from individuals living in the non-malaria region did not show an immune reaction with *An. gambiae *saliva (Figure 2). These results indicated that children living in the malaria endemic area had developed a specific IgG response against several proteins of *An. gambiae *saliva.

### Immunogenicity of major salivary proteins according to transmission season

**Figure 3:**
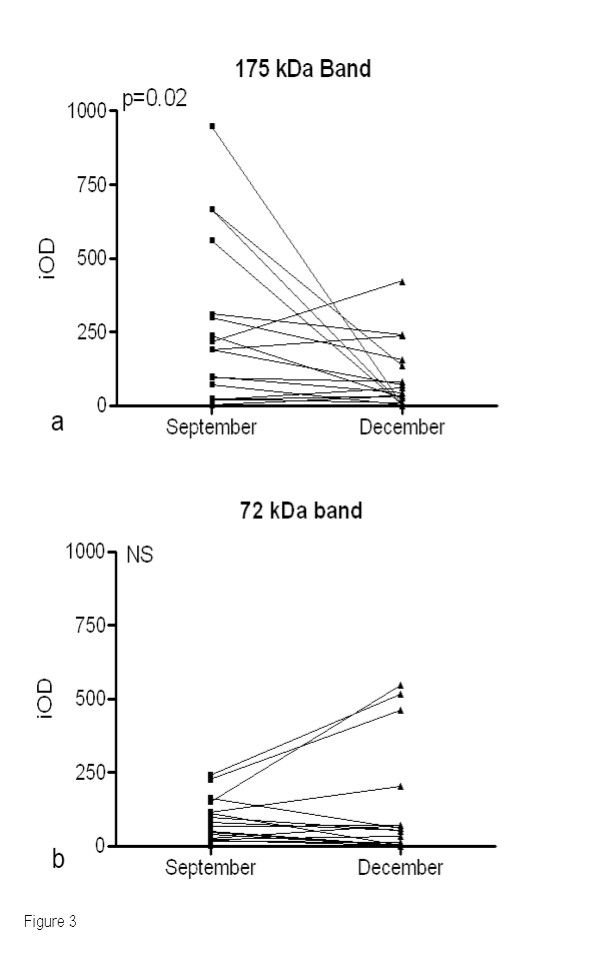
**Intensity analysis of individual IgG immunoblots of anti-saliva antibodies to *An. gambiae *in the peak- and post-season sera**. Intensity measurements of the 175 kDa (a) and 72-kDa (b) major bands were performed with the Scion image program using internal standard in September (peak) and December (post season). Results were reported as integrated optical density (iOD).

The intensity of both major bands (175 and 72 kDa) was measured for each paired serum from studied children. The comparison of intensities of the IgG Ab bands between peak- and post-malaria season sera (Figure 3) showed a significant decrease only for the 175 kDa Ab band (P = 0.02). In contrast, no significant differences were observed for the 72 kDa band. The seasonal intensities of these bands were compared according to data on the outcome of malaria attacks in these children. A significant seasonal decrease in the 175 kDa intensity was observed in children who suffered a malaria attack between September and December (n = 12; P = 0.03). No significant differences were observed for the 72 kDa band according to malaria status. No correlation was found between a decrease in the IgG Ab response and the age of the children (Spearman's correlation coefficient, R = -0.193, NS), nor according to the village of residency of the children.

## Discussion

The present study reported that children living in a malaria endemic area developed an IgG response against a limited number of *An. gambiae *salivary proteins. Interestingly, two major bands (72 and 175 kDa) were detected in all positive individuals. The results also showed a significant decrease in the intensity of the 175 kDa immunogenic band between the peak and the end of the malaria season.

This immunogenic band did not appear to be strongly expressed in saliva, as it did not match abundant proteins clearly visible on silver-stained gel. Along this line, sparse reports mentioned high molecular masses protein in *Anopheles *salivary glands; one is an anti-inflammatory factor described in *An. stephensi *salivary extracts [17], and the other a member from the SGS family which may play a role in malaria parasite invasion of salivary glands [22]. A putative assignation to the 72 kDa immunogenic band would be the apyrase, an inhibitor of platelet aggregation, or the maltase involved in sugar meal [23]. But the methodology used in the present study (1-D electrophoresis) appears limited to clearly associate one immunogenic band to one protein. However, less than 80 sequences have been defined as secreted products from *An. gambiae *saliva [7,8]. Discriminating proteomic tools could achieve the characterisation of these salivary antigens.

Surprisingly, only half of the children developed IgG Abs against *Anopheles *saliva. Previous studies in the field of allergy to mosquito bites have reported considerable variations of Abs response associated with mosquito species and population densities [24,25,16]. Here, no significant variation was observed according to villages: 40% of the children from the high exposed village were responders *versus *60% in the low exposed village. However, a spatial heterogeneity in malaria transmission is also observed elsewhere depending on the repartition of individuals at the village level [26]. Some of the children, because of the distance from major breeding sites, could have sparse contacts with *Anopheles *mosquito that may be insufficient to elicit anti-saliva Abs [18]. In addition, 20% of the population studied slept under a bednet also limiting the *Anopheles *bites [20]. Finally, the majority of the studies investigating human response to mosquito salivary proteins were performed in adults (over fifteen years old) [27,28] who have a longer history of exposure to mosquito bites and an immune response which could be slightly different than the one observed in young children [13]. The results presented here show variability between individuals in the immune response to salivary antigens. For example, two individuals presented a decrease in the 175 kDa band but an increase in the 72 kDa band intensity in December compared to September. Further studies integrating strong data on the evaluation of the intensity of *Anopheles *exposure are needed to determine whether one immunogenic band could reflect a "pattern" of specific exposure. Several epidemiological parameters, such as genetic background or the possibility of co-infections could also involved in the variations in immunogenicity observed between children [29-31].

The results presented here show a seasonal decrease in the intensity of immunogenic bands suggesting that the Ab response to *Anopheles *salivary antigens was transient, as previously observed in the same area [18]. As a matter of fact, it has been reported elsewhere that Ab against tick salivary antigens declines after a period of non-exposure [32] and clear seasonal difference in the anti-mosquito saliva Ab response has been described in Tanzania [11]. This transient character of the anti-saliva Ab response could be useful for the evaluation of *Anopheles *exposure, especially in areas with low intensity of exposure. Interestingly, the seasonal influence was significant for the 175 kDa band suggesting that this band could represent a good candidate for a marker of intensity of exposure to *Anopheles *bites. The results also showed a positive association with the intensity of immunogenicity for the 175 kDa band and the malaria morbidity in children over the same period. Indeed, the children developing malaria morbidity showed the higher intensity for this band in September which decreased during the malaria season. A previous study reported that the children presenting high IgG Abs in September are those will suffer from malaria attack within the next three months [18]. However, further studies including more subjects are necessary to confirm the association between anti-saliva Abs and malaria morbidity. This study described for the first time the presence of immunogenic bands against *Anopheles *saliva in children living in malaria endemic area. Few studies have described the anti-saliva Abs response to mosquito bite in children [13] whereas they are the main target for malaria parasite and other diseases transmitted by mosquito bites. Interestingly, the children who respond to *Anopheles *bites have common immunogenic bands suggesting that major common epitopes could be found in exposed populations. Theses results represent the first step toward the identification of immunogenic protein as a marker of exposure. Studies in other areas of malaria transmission and within a larger population were necessary to reinforce this hypothesis. But the methodology presented here, associating immunoblots and measurements of band intensity, appears very convenient for initial screening of molecules with an immunological and epidemiological interest. It provides qualitative and semi-quantitative data on the immunogenic components detected. Furthermore, such an approach could be applied to other vector-borne diseases which represent public health priorities in the developing world.

## Conclusion

The last ten years have witnessed a renewed interest in host/vector studies for controlling vector-borne diseases. Major efforts have been made in sequencing the genomes of most "killing" vectors [33] and the saliva proteome of the *Anopheles *vectors [7,23,34]. The physiological function of the salivary proteins has been confirmed for only a few of them. This work suggests the importance of the human/vector relationship to develop an integrated strategy for the evaluation of exposure to vector bites and thus the risk of malaria. These results suggest that salivary markers of exposure to mosquito bite may ultimately be identified.

## Authors' contributions

SC carried out immunoblot analysis, participated in the design of the study and drafted the manuscript.

FR conceived and coordinated the study and helped to draft the manuscript.

SD carried out protein preparation and immunoblots.

TN carried out mosquito saliva collection.

FXS participated in gel electrophoresis.

DB provided the scientific environment in Dakar and revised the manuscript.

FS participated in the conception and coordination of the study and helped to draft the manuscript.

All authors approved the final draft of the manuscript.
